# Scopemania

**Published:** 1875-02

**Authors:** Brewer Mattock

**Affiliations:** St. Paul, Minn.


SCOPEMANIA—*By Brewer Mattock, M.D., St. Paul, Minn.*—The designative or characteristic ornament of the surgery or office of the old-time physician or leech was, if we may trust the caricatures handed down to us, a stuffed alligator pendant from the ceiling; and in our mind's eye, we see the old worthy of former days wrapped in scientific contemplation, bewigged, with hands clasped, and eyes fixed upon this hanging suarian. A patient enters perchance, seeking advice and medicine; and we hear the old man's ponderous voice as he learnedly mouths over in the ears of his appreciative listener the set medical terms of the day. He discourseth of humors, of purulence, of pocks, of churnels, of disease of the womb, of sweats and pores, etc., etc.; and then he dispenses his complicated medicaments, of which he prides himself that they are composed of "rare and costly ingredients, full two score in number and very powerful." When the learned man ventures out to visit his patients, his designative badge is a cane, with snuff-attachment.

The representative medical man of the nineteenth century is not an old man; he is young or middle aged. He is essentially social, passably educated, dresses well, and behaves himself as a gentleman, when behavior is necessary; but his behavior never stands in the way of his enjoying his glass and his joke with those who helped him in his early days. As a rule, he never gets above himself and forgets old acquaintances, at least until he is firmly established with another set. He is partial to good horse-flesh, and insists on having his carriage re-varnished every spring. This is not merely a matter of pride with him; it makes him to be ready for a trade at a moment's notice. Your physician prides himself on never being cheated on a trade. His office is carpeted with dirty Brussels. His library is small and common-place. What the alligator was to the old doctor, the microscope is to the new. It is usually ostentatiously displayed, and is at once the center-piece and the chief object of attraction. It is sometimes flanked by a battery, or some sort of a spray producer, and always by a lot of uterine instruments. If the wall is not tucked about with photographs of patients before they were operated upon, it is because the space is occupied by diplomas or some other certificates. Maybe physicians are not members of such societies, notwithstanding the apparent interest.

In his office the physician of the present day is not one whit behind the old worthies in scientific conversation, and the superabundant use of medical terms. The terms have changed, however. The talk is now of organic lesions, textural changes, degeneration of tissues, displacements, inflammations, nervous irritations and catarrhs. He is debarred from enlarging in his remedies because he prescribes; but he loses not the opportunity of exhibiting his apparatuses as "necessities to a correct diagnosis."

When he goes abroad, he is recognized by the probangs, uterine sounds and speculums sticking out of his pockets—not ostentatiously are they displayed, but they are exhibited as if by inadvertence or accident. Characteristic physicians are like diseases; types change with the years, but the distinguishing peculiarities are easily recognized.

This much for prologue.

The tendency of the times seems to be the extravagant introduction of all sorts of new-fangled contrivances as mechanical aids to diagnosis and treatment; and the aspirant for medical honors sees advancement to professional success and standing only attainable by contriving, originating or improving upon some sort of an apparatus, appliance, or instrument.

To this we enter no protest. We say to the genius, go on inventing and improving, and maybe every five or ten years the profession will be indebted to some one. It would be strange if, out of ten thousand new mechanical combinations or contrivances, some one would not be valuable and lasting.

But here is where we would enter our protest. We object to strewing the way of young aspirants for professional standing with too much of this machinery; and we object to teachers laying too much stress in their teaching upon mechanical appliances, either in diagnosis or treatment, to the exclusion of the inculcation of sound professional principles.

Time was when a young man came home from his lectures with a sethescope and a pocket case, and commenced practice. Such a modest armentarium, we admit, was insufficient, but other instruments were sent for as needed. Now-a-days a young man that commences practice without an expensive microscope, an ophthalmascope, a laryngoscope, various stethescopes, several speculums, uterine knives, pessaries, sponge tents, urinometers, a respirator, etc., thinks himself unsupplied with the ordinary implements of his profession.

But we are answered, there is no law to prevent a young man from buying all that he wishes to or has money to purchase. We reply that there is necessity of teaching a student that high professional attainment can be reached without a knowledge of any, or at least but few of these things. We believe that the student should be first taught that his eyes, his ears, and his fingers, are for the present sufficient for diagnostic purposes.

Great is the man who can make a correct diagnosis with the thermometer, the stethescope, and the microscope; but greater is he that can form a diagnosis without them.

The student is taught that he must require from every female who complains of uterine distress that she submit to a visual or digital examination. How much moral and physical mischief has this cruel and indecent dictum occasioned! But the uterine specialists insist upon it, else their cry would be like the silversmiths at Ephesus—our occupation is gone.

Uterine examinations made with the frequency which is common at the present day are objectionable for numerous reasons. 1st. They are unnecessary, and in many instances do positive damage; they not only occasion pain, but oftentimes aggravate acute temporary irritations, and occasion positive suffering. 2d. They encourage the unnecessary use of the various appliances, they invite surgical interference and operations where no such operations are necessary, one of the most objectionable of which is the use of the uterine sound. How many abortions are yearly occasioned by the indiscriminate use of this infernal instrument? And how much unnecessary suffering does its use occasion? Then, if it were not for these examinations, how much less cauterizings would be practiced and pessaries introduced? The journals tell us of the severe uterine diseases cured, but do they ever touch upon the diseases occasioned as the result of the indiscriminate use of the speculum?

There seems to us to be a remarkable parallelism between the diseases of the throat and the diseases of the womb. The tissues are remarkably alike, and the diseases incident to both are much the same. We have congestions, irritations, granulations and ulcerations. We have enlargements, hypertrophy of the uvula and tonsils in the throat, as in the uterus. We have displacements, as muscular debility of the palate, and we have catarrhs of both structures; and the treatment usually adopted for the diseases incident to both is much the same.

It is but a few years since the profession, with an accord, commenced to cauterize the throat with nitrate of silver, and to sponge it out with probangs saturated with nitrate of silver. This practice has gone largely out of use, and gargles and spray have superseded it, when any medication is required, and in the vast majority of acute cases, we trust to rest of the parts and hygienic treatment alone. The time is not far distant, we trust, when diseases of the vagina and uterus will be treated as rationally.

We venture the assertion that there will yet appear one greater than Sims. The title of his work will be "the uterus hygienically and rationally considered." We venture the prophecy that he will advocate a non-meddlesome treatment. His remedies will be no more severe than injections, and his treatment will be largely constitutional. He will start with this fundamental doctrine: "Every female must expect, during her mature life, to suffer at times more or less uterine distress. Its amount and duration will depend largely upon herself."

Uterine disorders are, like dyspepsia, of almost universal frequency, and their cure must depend largely upon the afflicted individual. There are many chronic dyspeptics, just as there are many chronic uterine cases, which are practically incurable. Such patients usually are sensuous and senseless. Such patients add to our income, but subtract from our patience.

Our rather extended remarks upon the uterus are the result of witnessing, during an entire winter, and participating in, the treatment of the uterus, at one of the foreign female hospitals. A peculiarity of this hospital was that each of the four or five attending physicians—all men of eminence—treated uterine disorders differently from his colleagues. Each had a reputation to establish, and each worked upon a foundation of his own laying. One used pessaries, another caustics, another injections, and another treated his patients almost entirely constitutionally; and our experience was that the last named treatment was the most successful.

We add further, in closing this part of our subject, that at least two-thirds of the uterine examinations we witnessed that winter were wholly unnecessary; they could have been diagnosed without an examination.

What shall we say upon the ophthalmascope? We say frankly that it marks an era in the treatment of diseased eyes—in the hands of experienced operators. We think we are not out of the way in saying that there are in use in the United States at least ten thousand of these instruments.

We remember to have heard the late Professor Pope, of Illinois, speak as follows of this instrument: "I doubt if there are three men in the country who are perfectly familiar with the use of this instrument. As for me, it was three months before I could detect the least headway in its use." Its use is very much overrated, we think, and is applicable only to rare affections of the eye, and it is capable of doing a diseased eye much damage when improperly used; and yet how many doctors "couldn't live without it."

The laryngoscope is another instrument—valuable in the hands of the few. The sale of them is largely confined to young doctors. The instrument is harmless, but it tempts to the use of too much caustic.

The nasal douche has been of much value to many practitioners. But the harm that has resulted from its indiscriminate use has been incalculable. Yet there are "few families that can now do without them."

The thermometer no medical man should be without; in rare instances it is invaluable; but the indiscriminate use of it, we think, will result in its being laughed out of the profession in a few years. Let us suggest to the practitioner that, preceding its use, a glass of water be always called for, and it be washed, before and after using, in the presence of the patient and his friends. It is a desperately deceitful instrument, and one must not always send for the clergyman by its markings.

The spray producers and atomizers are of much value, and their use inaugurates a medical era.

The different kinds of instruments which cut automatically are, as a class, dangerous. We do not approve of cutting much by machinery.

Before graduating, we invented an elaborate instrument—a craniotome. We carried our model to several of the professors, some of whom complimented us upon our skill. At last we carried it to old Professor ———, who sat with his legs hoisted upon the stove. He looked it over very carefully, and replied: "A good thing, but I always use my jack-knife." If we are ever called upon to pierce a child's head, we shall use our pocket knife. The instrument still is a model—of misdirected ingenuity.

We would speak of but one class more of appliances, which, in most instances, are useless and mischievous—we refer to fracture apparatuses.

It may safely be stated that hardly any two fractures in our practice are similar. By this we do not mean the mere breaking of the bone, but the build, form, condition and age of the patient, as well as habits and surroundings. Take, for example, fractures of the thigh. One is a robust, extremely muscular, ignorant and stubborn laboring man. Another is a flabby, nervous old gentleman, who is constantly fidgeting and very intractable. Another is a man who is in good condition and scrupulously careful to obey directions. Another is a female; and the last a child. These patients, irrespective of the mere fracture, require almost as many different appliances, or rather modifications, of the one general principle of counter extension and immobility. We may treat one of these patients by simple counter extension and the sand-bags. Another must be strapped immovably to a long splint. Another must be treated by a molded splint or starch bandage; while we must humor the old gentleman with different appliances.

Mr. Surgeon A., B. or C., is a mechanic of the first class. He improvises or invents an apparatus for a particular case with great success. He therefore rushes to print and engraving, and introduces something new. The apparatus looks novel and reasonable, and is largely manufactured and sold. It falls into the hands of those who have not the ingenuity to apply it, or the judgment to decide upon its merits. The inventor himself may be never uses it again, because he never has another patient to whom it is applicable.

Now, the physician, young or old, who has no apparatus, is forced to treat a fracture on simple general principles. The simpler the appliance, the more care and watching is required, and the result better. Not only this, but the patient, understanding the theory and principle of treatment, interests himself in the cure. This he cannot do if the limb is locked up and hidden from view in an elaborate apparatus.

A case in point occurs to me. X—— sustained a fracture of the tibia and fibula. He was a young man of intelligence, and could have treated himself with a simple fracture box, and recovered without a trace of deformity, under the supervision of a wise physician. Unfortunately the young doctor locked it up in one of his preceptor's improved apparatus, and left it. The result was an overlapping of the bones and shortening—a scandalous result for such a fracture.

It should be remembered that the test of a first-class mechanic—an iron worker, if you please—is not his knowledge of a lathe, but of the file, the hammer, and cold chisel. A good carpenter is not tested by his knowledge of a mortising machine, but by the way he makes a mortice by hand. The test of a good surgeon is not how to apply apparatuses, but by getting along without them.

The same is true of diagnosing. Better is he who can diagnose a malignant growth without a microscope than he who relies only on his lens. Better be a good judge of the pulse by feel than be an expert manipulator of the sphygmograph. He who ties himself to an apparatus of any form, shape, or kind, allows another's invention to take the place of his own perception. If I use a mallet for percussing, I must discontinue the use of my finger-ends.

The same is true of the stethescope, but this instrument we can not so well dispense with.

Our conclusion of the whole matter is this: We would have the physician of to-day a man of judgment, of knowledge, and of brains. We would have the center-piece of his office a head, not a microscope nor an ostentatious display of instruments. We would have the young physician come home from his lectures with a well stored head, and not with a well stocked armentarium.—*Indiana Journal of Medicine.*

The Lloyd Map Company, noticed in a late number of this Journal, seems to be a swindle. The notice was based upon their autograph letter to this Journal.—*American Medical Weekly.* Our experience confirms the above.—Ed.

